# Reemergence of Scrub Typhus Associated with *Leptotrombidium akamushi* Mites and Karp-Like *Orientia tsutsugamushi* Genotype Bacteria, Japan, 2024–2025

**DOI:** 10.3201/eid3209.260646

**Published:** 2026-09

**Authors:** Motohiko Ogawa, Nobuhiro Takada, Takashi Katayama, Yuki Hashimoto, Kei Nagano, Hirotaka Komine, Yukihiro Akeda

**Affiliations:** National Institute of Infectious Diseases, Japan Institute for Health Security, Tokyo, Japan (M. Ogawa, Y. Hashimoto, Y. Akeda); University of Fukui, Fukui, Japan (N. Takada); Kanagawa Prefectural Institute of Public Health, Kanagawa, Japan (T. Katayama); Niigata City General Hospital, Niigata, Japan (K. Nagano); Yamagata University, Yamagata, Japan (H. Komine)

**Keywords:** *Orientia tsutsugamushi*, *Leptotrombidium akamushi*, scrub typhus, vector-borne infections, bacteria, rickettsia, zoonoses, large Japanese field mouse, *Apodemus speciosus*, seasonal variation, Japan

## Abstract

Scrub typhus is a miteborne infectious disease caused by *Orientia tsutsugamushi* bacteria. In Japan, most cases occur in spring and autumn and are transmitted by *Leptotrombidium pallidum* and *L. scutellare* mites; summer scrub typhus, transmitted by *L. akamushi* mites, is rarely reported. In August 2024, a pregnant woman had scrub typhus develop after attending a fireworks event in Niigata Prefecture, Japan, resulting in fetal death. A Vietnam-related Karp-like *O. tsutsugamushi* genotype, distinct from the Kato type usually associated with *L. akamushi* mites, was detected from patient samples. Field investigations at the site of the fireworks event revealed seasonal predominance of *L. akamushi* mites in summer. We detected *O. tsutsugamushi* sequences identical to the patient’s strain in 3 *L. akamushi* mite larvae and 1 field mouse, suggesting that *L. akamushi* mites were the source of human infection. Our findings suggest reemergence of summer scrub typhus associated with infected *L. akamushi* mites carrying a locally novel genotype.

Scrub typhus is a miteborne infectious disease caused by *Orientia tsutsugamushi* bacteria and transmitted by larval trombiculid mites of the genus *Leptotrombidium* (*1*)*.* Patients typically have an eschar develop at the site of the mite bite, accompanied by fever, rash, and elevated liver enzyme and C-reactive protein levels (*2*,*3*). Tetracycline-class antimicrobial agents are generally highly effective against scrub typhus, but delayed treatment can lead to severe disease and occasionally death.

In Japan, scrub typhus exhibits a bimodal seasonal distribution, with peaks occurring in spring and autumn (*2*). That seasonal pattern is largely determined by the life cycles of vector mite species. The principal vectors in Japan are *L. pallidum* and *L. scutellare* mites (*1*,*4*). Both species hatch in autumn, and their larvae parasitize vertebrate hosts, contributing to the autumn peak of disease incidence. *L. pallidum* larvae can overwinter and remain active until the following spring, thereby contributing to the spring peak of scrub typhus cases. Historically, summer scrub typhus, transmitted by *L. akamushi* mites, occurred in river basin environments of northern and central Japan, including major river systems in Akita, Yamagata, and Niigata Prefectures, such as the Shinano River Basin (*2*,*5*). However, summer scrub typhus is now rare, and only sporadic cases have been reported in recent decades, including a suspected Kato-type infection reported in Akita Prefecture in 2008 (*6*).

Different *Leptotrombidium* mite species are associated with specific *O. tsutsugamushi* serotypes. For example, *L. akamushi* mites are primarily associated with the Kato serotype, whereas *L. pallidum* and *L. scutellare* mites are associated with other major serotypes, including Gilliam, Karp, Kawasaki, and Kuroki (*2*,*7*,*8*). More recently, *L. palpale* mites have been proposed as a vector candidate for the Shimokoshi type *O. tsutsugamushi* bacteria (*9*). In Japan, reports of infections caused by the Kato serotype are rare, and the *O. tsutsugamushi* bacterial genotypes currently associated with *L. akamushi* mites remain poorly characterized in regions where summer scrub typhus was historically endemic and where that mite species was the principal vector.

In August 2024, a pregnant woman who had attended a fireworks event on a riverbank in Niigata Prefecture had scrub typhus develop, resulting in fetal death. She had no recent travel history outside Niigata Prefecture before symptom onset. *O. tsutsugamushi* bacteria were detected in the patient’s serum and placenta. Sequence analysis of the 56-kDa type–specific antigen (TSA) gene revealed a Karp-like genotype closely related to strains previously reported from Vietnam (*10*). The fireworks event, the suspected exposure site, was held in the Shinano River Basin, a region historically known for summer scrub typhus transmitted by *L. akamushi* mites. 

Detection of *O. tsutsugamushi* in larval mites can provide evidence of the direct source of human infection, and infection in rodents can provide indirect evidence of infected mites in the area. Therefore, we conducted a field investigation in the suspected exposure area to capture mites and field mice to determine *O. tsutsugamushi* infection and characterize the local maintenance of *O. tsutsugamushi* in mite vectors and their host species.

## Materials and Methods

**Figure 1:**
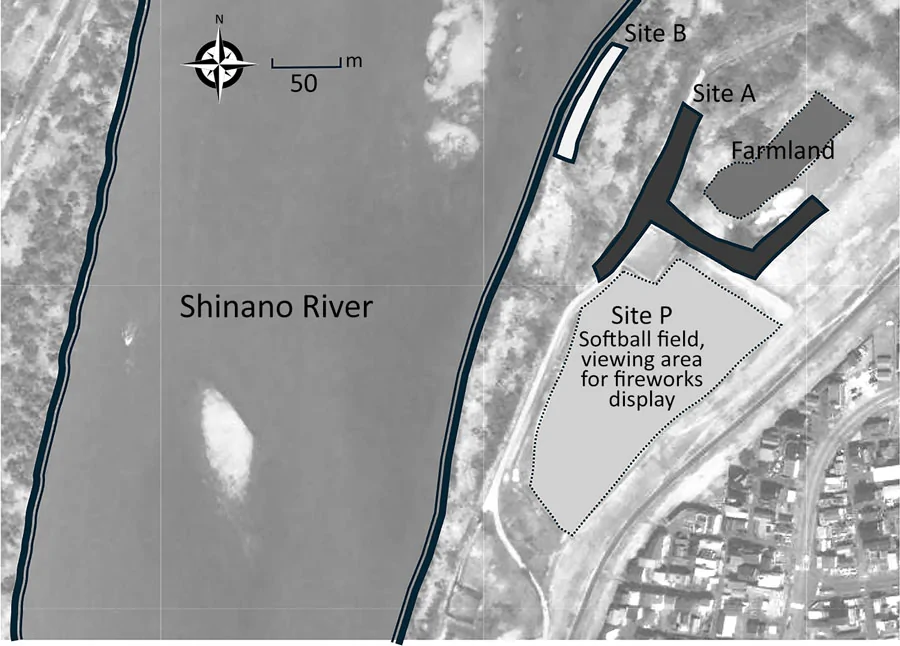
Study sites for investigation of reemergence of scrub typhus associated with *Leptotrombidium akamushi* mites and Karp-like *Orientia tsutsugamushi* genotype bacteria, Japan, 2024–2025. Map shows suspected exposure site along the Shinano River in Niigata Prefecture (P) and 2 sampling sites (A and B). Site A was located adjacent to farmland, and site B was located along the riverbank. Sites A and B are within 200 m of the patient’s suspected exposure site.

We conducted a field investigation at the suspected exposure site along a riverbank in Nagaoka City, Niigata Prefecture, Japan (37°27.283′N, 138°50.269′E). We selected 2 sampling sites within ≈200 meters of the suspected exposure site: site A was an area of dense bushy vegetation adjacent to farmland, and site B was an area of dense bushy vegetation immediately adjacent to the river’s edge (Figure 1). Both sites provided suitable habitats for trombiculid mites and small mammal hosts, including *Apodemus* spp. field mice.

We conducted field surveys at 1 or both sites during June, July, August, and November 2025; site A was not sampled in August. We used Sherman traps to capture field mice and set ≈50 traps per night across both sites. We anesthetized captured field mice and collected blood by cardiac puncture for serologic testing before humanely euthanizing the mice. We then collected spleen samples for molecular detection. We carefully removed attached larval trombiculid mites from field mice for mite species identification and *O. tsutsugamushi* detection. The Institutional Animal Care and Use Committee of the National Institute of Infectious Diseases approved all animal procedures (approval no. 125159).

### Mite Identification

We morphologically identified mites under a stereomicroscope, according to standard taxonomic methods (*11*). Because *L. akamushi* mites have rarely been reported in recent field surveys, we performed molecular identification on selected specimens on the basis of the cytochrome oxidase I (*COX1*) gene (*12*) to verify species identification. We used PrepMan Ultra Sample Preparation Reagent (Thermo Fisher Scientific, https://www.thermofisher.com) to extract DNA from individual mites. 

### Detection of *O. tsutsugamushi* in Mice and Mites

We tested mouse serum samples for 6 *O. tsutsugamushi* antibody serotypes by using an indirect immunofluorescence assay (*13*) and considered titers >80 positive. We extracted DNA from spleen samples by using a FavorPrep Tissue Genomic DNA Extraction Mini Kit (Favorgen Biotech Corp., https://www.favorgen.com). We used real-time PCR targeting the 16S rRNA gene (*14*) on DNA extracted from field mouse spleens and engorged mites to screen for *O. tsutsugamushi*. For samples that tested positive or equivocal, we conducted further analysis by using nested PCR targeting the 56-kDa TSA gene (*15*). We sequenced amplicons and performed phylogenetic analysis by using the neighbor-joining method in MEGA X version 11.01.3 (https://www.megasoftware.net) with 1,000 bootstrap replicates (*16*).

## Results

### Detection of *O. tsutsugamushi* in Mice

**Figure 2:**
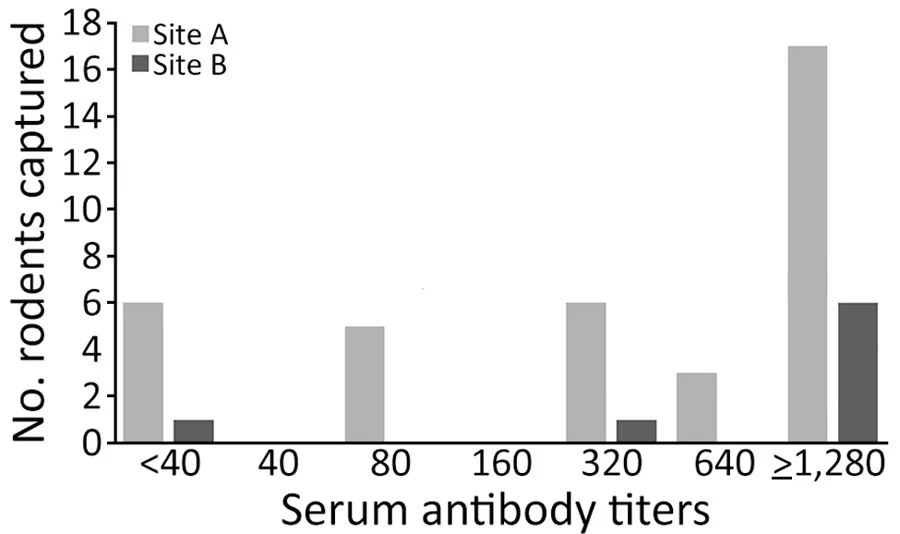
Antibody titers from field mice in a study of reemergence of scrub typhus associated with *Leptotrombidium akamushi* mites and Karp-like *Orientia tsutsugamushi* genotype bacteria, Japan, 2024–2025. We tested serum samples from large Japanese field mice (*Apodemus speciosus*) by indirect immunofluorescence assay against 6 *O. tsutsugamushi* bacterial serotypes. Titers >80 were considered positive.

We captured a total of 54 field mice, all of which were large Japanese field mice (*Apodemus speciosus*). Serum samples suitable for serologic testing were obtained from 45 mice (37 from site A and 8 from site B). From site A, 31 (84%) of 37 tested field mice were *O. tsutsugamushi*–seropositive. In site B, 7 (88%) of 8 tested field mice were *O. tsutsugamushi*–seropositive (Figure 2). From site A, PCR genotyping revealed Japanese Gilliam–type strains of *O. tsutsugamushi* bacteria in field mice collected during June, July, and November, and we detected a Karp-type strain in 1 field mouse collected in June. At site B, we detected Japanese Gilliam–type strains in 1 field mouse in June.

### Mite Abundance and Identification

**Figure 3:**
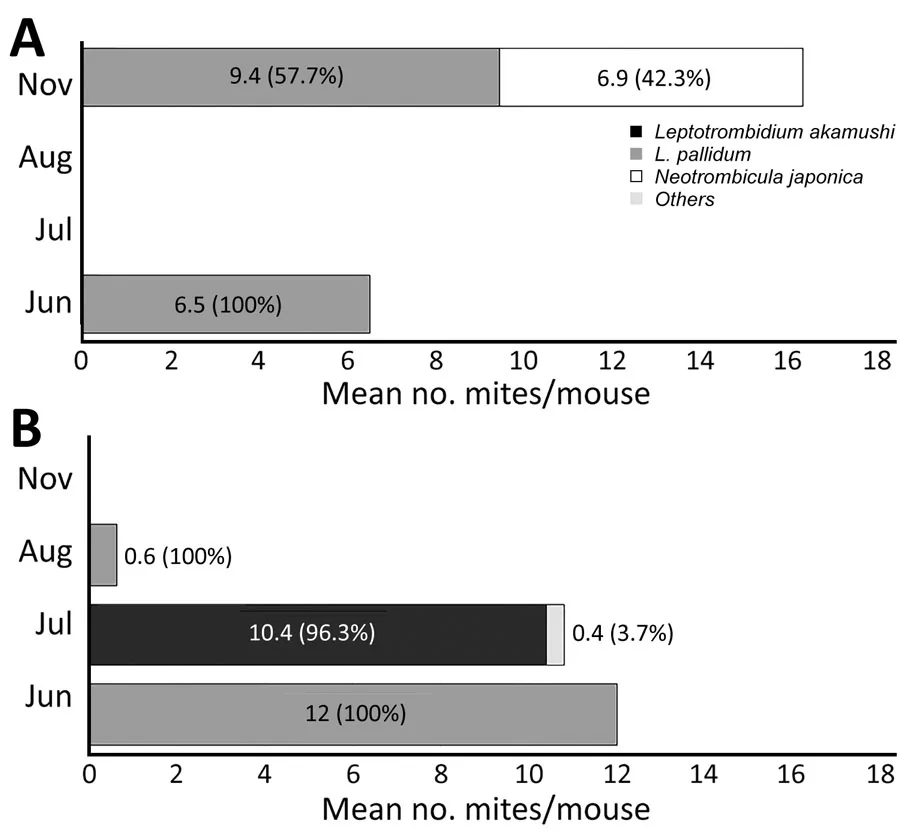
Seasonal abundance and species composition of trombiculid mites collected from field mice in a study of reemergence of scrub typhus associated with *Leptotrombidium akamushi* mites and Karp-like *Orientia tsutsugamushi* genotype bacteria, Japan, 2024–2025. A) Site A, near a tract of farmland; B) site B, near the riverbank. Graphs show mean numbers of mites per mouse and species composition for each sampling period. Mite species composition differed markedly between the sites: *L. pallidum* was predominant at site A in June (early summer) and November (autumn), whereas *L. akamushi* was predominant at site B in July (summer). *Neotrombicula japonica* and other mite species were also detected.

The composition and seasonal abundance of mites collected from field mice differed markedly between the 2 sampling sites. From site A, we detected mites in November (mean 16.3 mites/mouse) and June (mean 6.5 mites/mouse) but not in July (Table).  We conducted no sampling at site A in August. All mites collected in June were *L. pallidum*, whereas 57.7% of mites collected in November were *L. pallidum* and 42.3% were *Neotrombicula japonica* (Figure 3). At site B, all mites collected in June and August were *L. pallidum*, whereas *L. akamushi* accounted for 96.3% of mites collected in July (Figure 3).

**Table:** Number of mice and mites collected per site in an investigation of reemergence of scrub typhus associated with *Leptotrombidium akamushi* mites and Karp-like *Orientia tsutsugamushi* genotype bacteria, Japan, 2024–2025*

Collection site and month collected	Total no. mice captured	No. mites collected†	
		Total no. collected	Mean no./mouse
Site A			
June	20	130	6.5
July	11	0	0
August	NS	NS	NS
November	7	114	16.3
Site B			
June	2	24	12
July	5	54	10.8
August	8	5	0.6
November	1	0	0

*Sampling sites were within ≈200 meters of the suspected exposure site in Nagaoka City, Niigata Prefecture, Japan (37°27.283′N, 138°50.269′E). Site A was dense bushy vegetation adjacent to farmland; and site B was dense bushy vegetation immediately adjacent to the Shinano River. NS, not sampled. †Mite species included *L. akamushi*, *L. pallidum*, *Neotrombicula japonica*, and other unidentified species.

**Figure 4:**
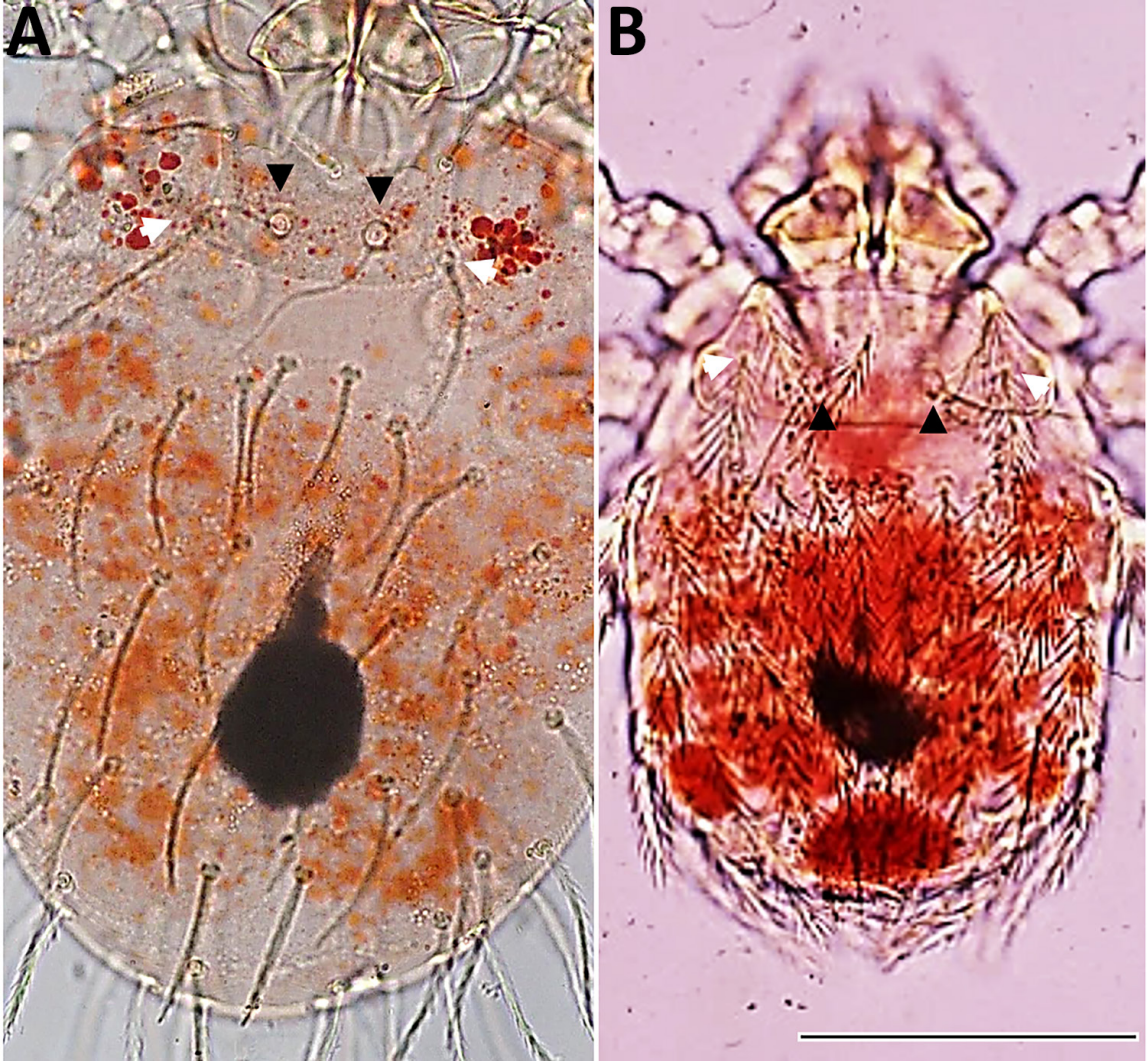
Comparison of engorged larval mites from a study of reemergence of scrub typhus associated with *Leptotrombidium akamushi* mites and Karp-like *Orientia tsutsugamushi* genotype bacteria, Japan, 2024–2025. A) *L. akamushi* larva; B) semi-engorged *L. pallidum* larva. Larvae were collected from field mice in Niigata Prefecture at the site of probable patient *O. tsutsugamushi* infection. *L. akamushi* can be distinguished from *L. pallidum* by the position of the sensillary setae (black arrows), which arise anterior to the posterolateral setae of the scutum, and by the presence of 8 dorsal idiosomal setae in a transverse row (white arrows). Those morphological characteristics were consistent with the molecular identification on the basis of *COX1* sequence analysis. Scale bar indicates 100 μm; both images are at the same magnification.

Representative mite specimens showed the characteristic position of the sensillary setae anterior to the posterolateral setae and 8 dorsal idiosomal setae, consistent with *L. akamushi* mites (Figure 4). Of *L. akamushi* 52 mites analyzed, 24 yielded *COX1* sequences confirming species identification. The other 28 specimens did not yield reliable sequences, likely because of the extremely small body size of larvae and variable DNA quality. However, all specimens exhibited morphologic characteristics consistent with established diagnostic criteria for *L. akamushi* mites under stereomicroscopic examination. Together with the molecular results, those findings indicate the presence of *L. akamushi* mites at the study site.

### Phylogenetic Analysis of *O. tsutsugamushi* Strains

We identified Japanese Gilliam–type *O. tsutsugamushi* strains in 5 field mice collected at site A in June, 1 field mouse collected in July, and 2 field mice collected in November. At site B, we identified the same strain in 1 field mouse and 1 mite collected in June. We identified a Karp-like strain of *O. tsutsugamushi* in 1 field mouse and 3 *L. akamushi* larvae collected at site B in July. *COX1* analysis confirmed the larvae as *L. akamushi* species mites.

**Figure 5:**
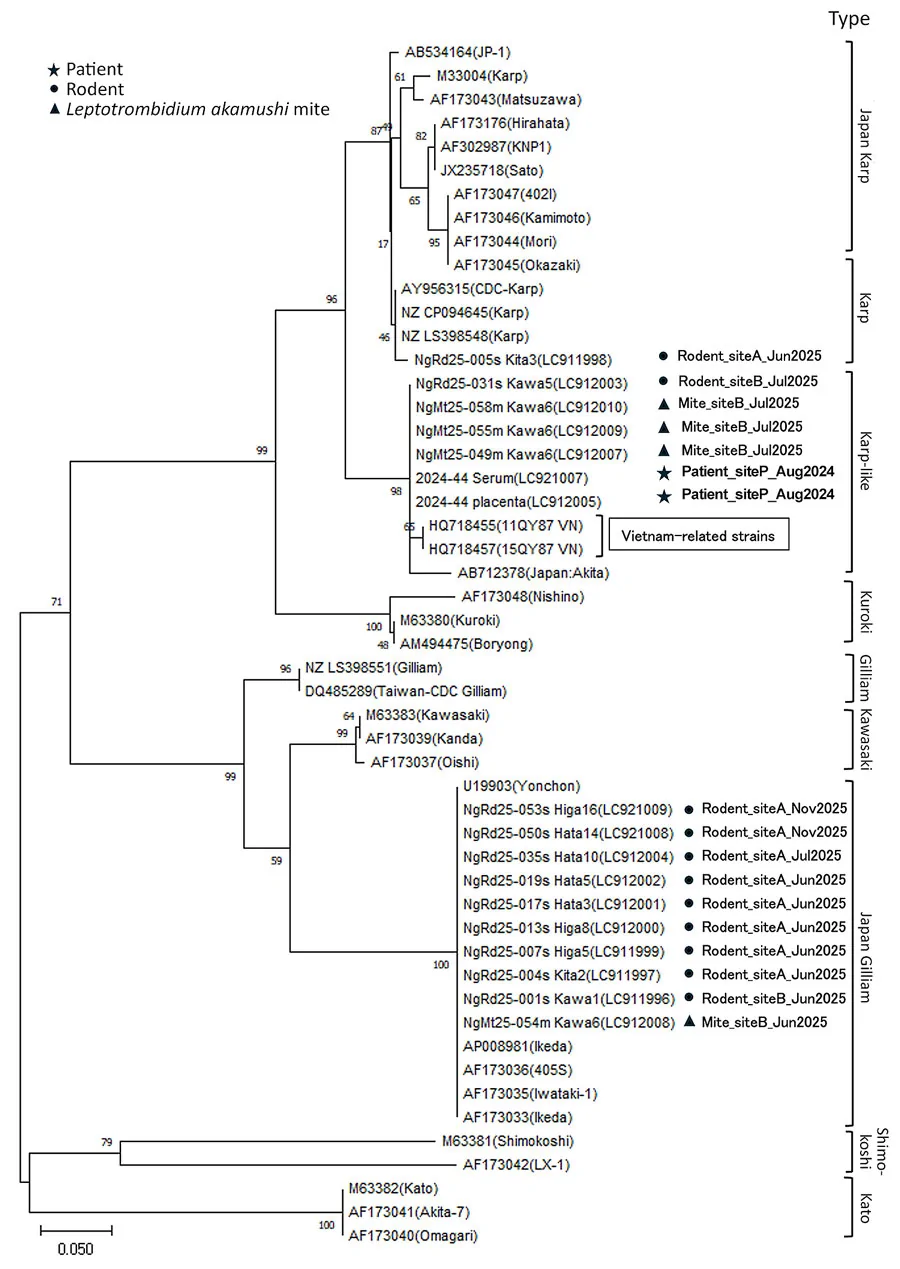
Phylogenetic relationships of *Orientia tsutsugamushi* strains detected in field mice, mites, and a human patient from a study of reemergence of scrub typhus associated with *Leptotrombidium akamushi* mites and Karp-like *O. tsutsugamushi* genotype bacteria, Japan, 2024–2025. Phylogenetic analysis was performed using the neighbor-joining method on the basis of an ≈420-bp region of the 56-kDa type–specific antigen gene. Sequences obtained from the placenta specimen, patient serum, 1 field mouse, and 3 *L. akamushi* mite larvae clustered together within the Karp-like lineage and were identical within the analyzed region. The placenta sequence (GenBank accession no. LC912005) was also identical over a 959-bp region to 2 *L. akamushi* mite–derived sequences (GenBank accession nos. LC912009 and LC912010). Reference strains representing major genotypes, including Kato and Karp-related strains, are included for comparison. *O. tsutsugamushi* bacterial sequences from rodents were all obtained from large Japanese field mice (*Apodemus speciosus*). Scale bar indicates nucleotide substitutions per site.

The Karp-like strain of *O. tsutsugamushi* we detected was more closely related to strains from Vietnam than to the Kato type historically associated with *L. akamushi* mites in Japan. The longest 56-kDa TSA gene sequence (959 bp) we obtained was from the placenta specimen (GenBank accession no. LC912005). That sequence showed the highest nucleotide identity (98.5%) to 2 previously reported strains from Vietnam (GenBank accession nos. HQ718457.1 [945 bp] and HQ718455.1 [959 bp]) (Figure 5). Related Karp-like sequences have been identified elsewhere in Asia, but those 2 strains from Vietnam had the highest sequence similarity to sequence LC912005. In addition, the 959-bp sequence obtained from the placenta specimen was identical to 2 other sequences (GenBank accession nos. LC912009 and LC912010) obtained from 2 *L. akamushi* mite larvae. The nucleotide sequences of an ≈420-bp region were identical among the placenta specimen (GenBank accession no. LC912005), patient serum sample (accession no. LC921007), 1 field mouse spleen sample (accession no. LC912003), and 3 *L. akamushi* larvae (accession nos. LC912007, LC912009, and LC912010). Phylogenetic analysis on the basis of that 420-bp region placed all samples within the same Karp-like cluster (Figure 5).

The *O. tsutsugamushi*–positive larvae were engorged *L. akamushi* larvae attached to field mice at the time of collection. However, the spleens of the field mouse hosts to which those larvae were attached tested negative for *O. tsutsugamushi* by PCR. Because trombiculid larvae feed only once on vertebrate hosts, larvae that acquire *O. tsutsugamushi* from an infected host cannot subsequently transmit the pathogen to another host. Moreover, horizontally acquired infection is not efficiently maintained across generations (*8*,*17*). Therefore, detection of *O. tsutsugamushi* in engorged larvae attached to PCR-negative field mice strongly suggests that those larvae acquired the pathogen before attaching to the mice, likely through vertical transmission; therefore, *L. akamushi* could be the source of human infection in this instance. Furthermore, sequence analysis showed that the nucleotide sequences of the *O. tsutsugamushi* bacterial strain in the larvae were identical to those detected in the patient’s serum and placenta, providing strong evidence that human infection was likely caused by bites of infected *L. akamushi* larvae during the fireworks event on the riverbank.

## Discussion

Our findings demonstrate marked seasonal differences in mite species abundance and composition within a limited area along a riverbank in Niigata Prefecture and identified *L. akamushi* as the predominant mite species at the time and location where the patient is suspected to have been exposed. Detection of the same genotype of *O. tsutsugamushi* in *L. akamushi* mites, field mice, and the patient suggests that human infection occurred at this site.

The marked predominance of *L. akamushi* in July suggests that this species is strongly associated with the summer occurrence of *O. tsutsugamushi* scrub typhus at this site. Historically, *L. akamushi* mites have been recognized as the principal vector of summer scrub typhus in northern Japan, particularly in river basin environments. The contrasting predominance of *L. pallidum* mites at site A and *L. akamushi* mites at site B might reflect differences in local environmental conditions, although the specific factors responsible remain unclear.

Of note, we identified engorged *O. tsutsugamushi*–positive larvae attached to field mice whose spleens tested *O. tsutsugamushi*–negative by PCR, indicating that the larvae acquired the pathogen before attaching to their hosts. Because trombiculid larvae feed only once on vertebrate hosts and horizontally acquired infection is not efficiently maintained across generations, only larvae that already harbor *O. tsutsugamushi*, presumably through vertical transmission, can serve as the source of human infection. Those findings suggest that *O. tsutsugamushi* is maintained primarily within mite populations and that infected larval mites are the principal source of human infection, rather than vertebrate hosts serving as amplification reservoirs.

The strain we identified was a Vietnam-related Karp-like genotype, distinct from the Kato type historically associated with *L. akamushi* mites, suggesting the presence of a previously unrecognized lineage associated with this vector species in Japan. That finding demonstrates that *L. akamushi* mites are not associated exclusively with the Kato genotype, as previously thought, but can also harbor genetically distinct lineages, including Vietnam-related Karp-like strains.

In summary, the high seroprevalence in field mice at both survey sites suggests widespread circulation of *O. tsutsugamushi* and infected vector mites within the study area. Collectively, our findings provide evidence for the re-emergence of summer scrub typhus associated with infected *L. akamushi* mites and highlight the need for continued vector surveillance and molecular *O. tsutsugamushi* bacterial surveillance to clarify scrub typhus risk in Japan.
